# Gengnianchun Extends the Lifespan of *Caenorhabditis elegans* via the Insulin/IGF-1 Signalling Pathway

**DOI:** 10.1155/2018/4740739

**Published:** 2018-02-18

**Authors:** Fanhui Meng, Jun Li, Yanqiu Rao, Wenjun Wang, Yan Fu

**Affiliations:** ^1^Department of Gynecology, The First Hospital of Jilin University, Changchun, China; ^2^Department of Integrated Traditional Chinese Medicine and Western Medicine, Obstetrical and Gynecological Hospital, Fudan University, Shanghai, China

## Abstract

Gengnianchun (GNC), a traditional Chinese medicine (TCM), is believed to have beneficial effects on ageing-related diseases, such as antioxidant properties and effects against A*β*-induced toxicity. We previously found that GNC extended the lifespan of *Caenorhabditis elegans*. However, the mechanism underlying this effect was unclear. In this study, we further explored the mechanisms of GNC using a *C. elegans* model. GNC significantly increased the lifespan of *C. elegans* and enhanced oxidative and thermal stress resistance. Moreover, chemotaxis increased after GNC treatment. RNA-seq analysis showed that GNC regulated genes associated with longevity. We also conducted lifespan assays with a series of worm mutants. The results showed that GNC significantly extended the lifespan of several mutant strains, including eat-2 (ad465), rsks-1 (ok1255), and glp-1 (e2144), suggesting that the prolongevity effect of GNC is independent of the function of these genes. However, GNC failed to extend the lifespan of daf-2 (e1370), age-1 (hx546), and daf-16 (mu86) mutant strains. Our findings suggest that GNC extends the lifespan of *C. elegans* via the insulin/IGF-1 signalling pathway and may be a potential antiageing agent.

## 1. Introduction

Ageing is becoming a major international concern that presents formidable challenges to healthcare systems. The effects of biological ageing contribute to a considerable share of human suffering. Ageing is proposed as a major risk factor for various diseases, such as cancer, diabetes, and cardiovascular and neurodegenerative disorders, including Alzheimer's, Huntington's, and Parkinson's diseases [1]. As the global population lives longer, there is an increasing need for strategies to improve the ageing process and ageing-related diseases.

Lifespan is regulated by various signalling pathways and transcription factors, such as insulin/insulin-like growth factor (IGF) and germline signalling and target of rapamycin (TOR) pathways [2]. With the elucidation of multiple signalling pathways that affect ageing, many strategies that prolong lifespan and improve health are being explored. Among which, application of plant-derived drugs to interfere with the ageing process has received an increasing amount of attention. For example, natural thioallyl compounds have been shown to increase oxidative stress resistance and lifespan [3], and chlorogenic acid extended the lifespan of *C. elegans* via the insulin/IGF-1 signalling pathway [4].

Gengnianchun (GNC), a traditional Chinese medicine (TCM) formula, is composed of *Radix Rehmanniae*, *Rhizoma Coptidis*, *Radix Paeoniae Alba*, *Rhizoma Anemarrhenae*, *Cistanche salsa*, *Radix Morindae Officinalis*, *Poria*, *Epimedium Brevicornum*, Cortex Phellodendri Amurensis, *Fructus Lycii*, *Semen Cuscutae*, and Carapax et plastrum Testudinis. GNC has been used in the clinic to alleviate declining functions related to ageing because of its kidney/liver-tonifying effect. In previous studies, GNC was shown to exert antiaging effects by modulating the hypothalamus-pituitary-ovary axis, increasing the estradiol receptor (ER) level in the pituitary gland and ovaries and increasing the concentration of *β*-EP in the hypothalamus [5]. In Yuan's research, GNC significantly increased the hydroxyproline (HYP) level and the SOD activity in a skin-aging model [6]. More recently, we found that GNC enhanced resistance to oxidative stress and ameliorated *β*-amyloid peptide toxicity in vivo [7, 8]. These findings suggest that GNC may delay the ageing process.

*Caenorhabditis elegans* is an excellent model for ageing research because of its short lifespan, small size, and genetic tractability. The ageing signalling pathways are conserved from yeast to worms and mammals, such as mice and human beings [9]. Furthermore, *C. elegans* possesses genes homologous to 60–80% of the human genome [10]. *C. elegans* has been used as a well-characterized experimental system to screen chemicals with longevity modulatory effects [11, 12].

In the present study, we used *C. elegans* to investigate the mechanism by which GNC impacts the ageing process. We found that GNC significantly extended the lifespan of the nematode *C. elegans* and delayed the age-related decline in chemotaxis ability. Subsequent genetic analysis revealed that GNC extends the lifespan of *C. elegans* through the insulin/IGF-1 signalling pathway.

## 2. Materials and Methods

### 2.1. *C. elegans* Strains and Maintenance

Wild-type *C. elegans* N2 (Bristol) and the mutant strains CB1370 daf-2 (e1370), TJ1052 age-1 (hx546), CF1038 daf-16 (mu86), DA465 eat-2 (ad465), CF1903 glp-1 (e2144), and RB1206 rsks-1 (ok1255) were used in this study. All nematode strains and the *E. coli* strain OP50 were obtained from the Caenorhabditis Genetics Center (CGC) at the University of Minnesota (Minneapolis, MN, US). Worms were maintained at 20°C on solid nematode growth medium (NGM) plates seeded with *E. coli* OP50; for CF1903 glp-1 (e2144) cultures, animals were transferred to 25°C after 24 hours at 20°C; for CB1370 daf-2 (e1370) cultures, the nematodes were maintained at 16°C for 3 days and then transferred to 20°C until the desired stage of development was reached.

### 2.2. Preparation of the GNC Formula

The GNC formula is a conventional product for clinical use that contains 12 crude herbs. The GNC formula is composed of *Radix Rehmanniae*, *Rhizoma Coptidis*, *Radix Paeoniae Alba*, *Rhizoma Anemarrhenae*, *Cistanche salsa*, *Radix Morindae Officinalis*, *Poria*, *Epimedium Brevicornum*, Cortex Phellodendri Amurensis, *Fructus Lycii*, *Semen Cuscutae*, and Carapax et plastrum Testudinis. Preparation of the GNC formula was described in our previous reports [7, 8]. In this study, we used a mixture of the water extracts of the 12 crude herbs (the exact proportion of each herb was shown in Supplementary Table 1), which were purchased from Tianjiang Pharmaceutical (Jiangyin, China). These water extracts were manufactured with rigid quality control protocols. Furthermore, the water extracts used in this study were produced in the same batch.

### 2.3. Lifespan Assays

Lifespan assays were performed as previously described [7]; 1-2 age-synchronized day 1 adult nematodes were added to each well of 96-well plates containing 80 *μ*L of S-complete liquid medium with GNC (3.94 mg/mL) or vehicle (H_2_O). *E. coli* OP50 was added to the medium. Worms were cultured at 20°C. Survival was assessed every other day until death. The animals were scored as dead when they failed to respond to touching with a platinum wire pick. Approximately 60 worms were assigned to each group, and consistent results were obtained from independent experiments.

### 2.4. Oxidative and Heat Stress Resistance Assays

In the stress resistance assay, age-synchronized day 1 adult worms were treated with GNC (3.94 mg/mL) in S-complete liquid medium for 48 h; then, the worms were subjected to acute oxidative stress with H_2_O_2_ (16 mM) in M9 buffer for 4 h, chronic oxidative stress with paraquat (5 mM) for 9 days, or acute heat stress at 35°C for 10 h. Survival was determined by touch-provoked movement as described above.

### 2.5. Chemotaxis Assays

The chemotaxis assays were performed as described previously with some modifications [13]. Briefly, age-synchronized day 1 adult worms were incubated in S-complete liquid medium with control or GNC for 48 h; before the chemotaxis assay, worms were washed three times. In this study, 9 cm diameter agar plates containing 10 mL of 2.0% agar solution (1 mM MgSO_4_, 5 mM KH_2_PO_4_, pH 6.0 adjusted with KOH, 20 g/L agar) were prepared. Ten microliters of attractant (0.5 M sodium acetate) was placed twice onto the centre of region A (10 *μ*L of water was placed on the opposite side, region C) 15–18 h and 3 h before each assay. Each assay was started by placing approximately 30 washed worms at the centre of the assay plate. Shortly before the assay, 1.0 *μ*L of 1 M sodium azide solution was spotted onto the centre of regions A and C to anaesthetize the worms. The worms were scored after 90 min to calculate the chemotaxis index (CI) using the following formula: CI = (N_A_ − N_C_)/(N_A_ + N_C_). The experiments were independently repeated at least three times.

### 2.6. Reproduction Assays

The effect of GNC on fecundity was assessed by determining the total number of progeny produced per worm after exposure to GNC following the standard protocol [14]. The L4 larvae were individually transferred to a fresh plate previously spotted with GNC or control each day until the end of the reproductive period. The offspring of each worm were counted at the L2 or L3 stage.

### 2.7. RNA Sequencing (RNA-Seq) Analysis

Gene expression of *C. elegans* wild-type N2 treated with GNC (3.94 mg/mL) and controls at the same age (old worms, 22 days old) was analysed using RNA-seq. Total RNA was extracted using RNAiso Plus Total RNA extraction reagent (TaKaRa, China) and purified with an RNAClean XP Kit (Beckman Coulter Inc., Kraemer Boulevard Brea, CA, USA) and an RNase-Free DNase Set (Qiagen, GmBH, Germany). The library construction and sequencing were performed at Shanghai Biotechnology Corporation with an Illumina HiSeq 2500 instrument (Illumina, USA). The sequence quality of the data sets was assessed using an Agilent Bioanalyzer 2100 (Agilent Technologies, Santa Clara, CA, USA). Transcript levels were estimated using fragments per kilobases per million reads (FPKM) values to allow us to compare different genes or samples. The up- or downregulated genes were identified by filtering the RNA-seq data with the following cut-off: twofold change in the expression level and a false discovery rate (FDR) analogue of *p* value less than 0.05. All analyses were performed at Shanghai Biotechnology Corporation (Shanghai, China). Gene expression data was deposited in the Gene Expression Omnibus (GEO) database with the accession number GSE98195.

### 2.8. Validation of the RNA-Seq Results via Quantitative Real-Time PCR (qPCR)

To validate the RNA-seq results, we performed qPCR analyses on a qTOWER 2.2 Real-Time PCR System (Analytik Jena AG, Thuringia, Germany). The specifically designed primers are listed in Table 1. The relative gene expression levels were calculated using the 2^−ΔΔCT^ method with the gene act-4 as the internal control. The experiment was repeated in triplicate.

### 2.9. Statistical Analysis

GraphPad Prism software 6.0 was used for statistical analyses. For the lifespan assay, a Kaplan-Meier survival analysis was conducted, and *p* values were calculated using a log-rank test. Student's *t*-test was performed to compare two data sets. One-way analysis of variance (ANOVA) with Duncan's test was used to compare multiple groups. *p* < 0.05 was considered statistically significant.

## 3. Results

### 3.1. GNC Enhanced the Lifespan and Stress Resistance of *C. elegans*

**Figure 1:**
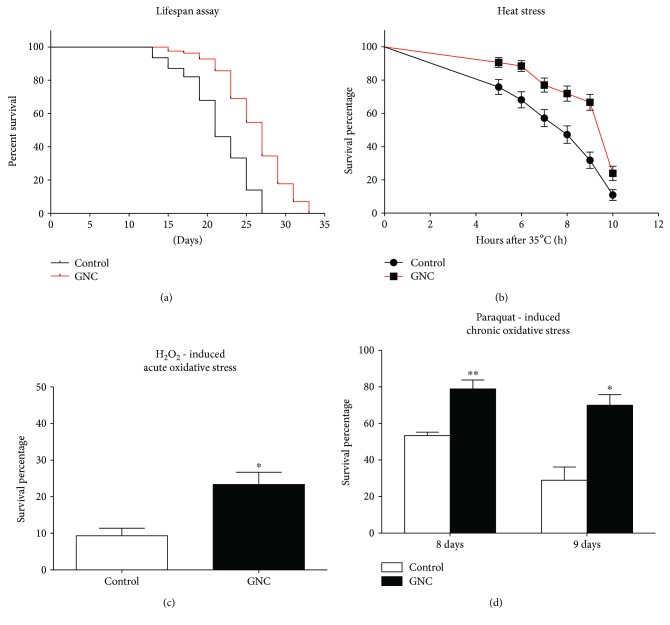
GNC treatment extended the lifespan and enhanced resistance to heat stress and oxidative stress in *C. elegans* N2. (a) The lifespan of *C. elegans* N2 at 20°C. (b) Survival percentage of N2 nematodes subjected to acute heat stress. (c) Survival percentage of N2 nematodes subjected to H_2_O_2_-induced oxidative stress. (d) Survival percentage of N2 nematodes subjected to paraquat-induced oxidative stress. (b–d) After treatment with 3.94 mg/mL GNC for 48 h, nematodes were dipped into 16 mM H_2_O_2_ for 4 h or 5 mM paraquat for 9 consecutive days or moved into a 35°C incubator, and then, the surviving worms were counted. The data are displayed as the mean ± SEM. ^∗^ *p* < 0.05; ^∗∗^ *p* < 0.01.

In our previous study, we found that 3.94 mg/mL GNC significantly increased the lifespan of *C. elegans* [7]. Increased longevity was correlated with tolerance to stresses. In this study, we tested whether GNC-treated *C. elegans* N2 showed increased resistance to oxidative and heat stresses. H_2_O_2_ and paraquat were used as acute and chronic oxidative stress inducers, respectively. Under H_2_O_2_-induced oxidative stress, GNC (3.94 mg/mL) pretreatment provided significant protection, with 23.33% versus 9.33% (control) of the worms surviving (Figure 1(c)). Under paraquat-induced oxidative stress, the survival rate of worms pretreated with GNC (3.94 mg/mL) was 78.89% versus 53.33% of the control (8 days) and 70.00% versus 28.89% (9 days) (Figure 1(d)). Under acute thermal stress at 35°C, the overall survival increased significantly with GNC (3.94 mg/mL) pretreatment compared with the control (*p* < 0.0001), with 23.96% versus 10.99% (control) of the worms surviving (10 hours) (Figure 1(b)).

### 3.2. GNC Increased the Chemotaxis Ability of Worms

**Figure 2:**
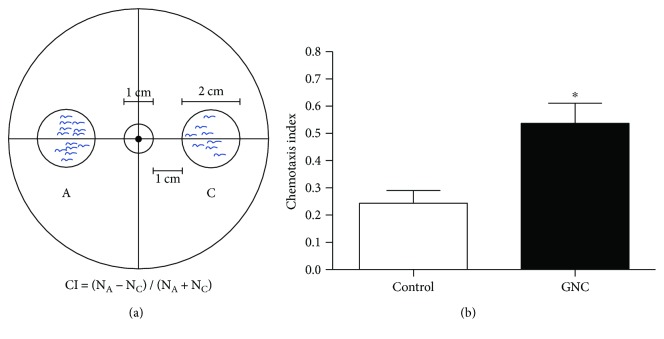
GNC increased the CI of *C. elegans* N2. (a) Schematic diagram of the chemotaxis assay. Chemoattractant was placed at the centre of region A and allowed to form a concentration gradient. Worms were placed at the centre of the plate. (b) The CI of *C. elegans* N2 treated with vehicle or GNC (3.94 mg/mL). Quantitative values are shown as the mean ± SEM of three independent measurements. ^∗^ *p* < 0.05.

Chemotaxis behaviour declines with ageing in *C. elegans* [15]. Therefore, to investigate the effects of GNC on responses to sensory stimuli, we examined the CI of day 3 worms. In GNC-treated worms, the chemotaxis behaviour was significantly improved, with 0.54 versus 0.24 (control) of CI (*p* = 0.0289) (Figure 2).

### 3.3. GNC Had No Effect on the Fecundity of *C. elegans*

**Figure 3:**
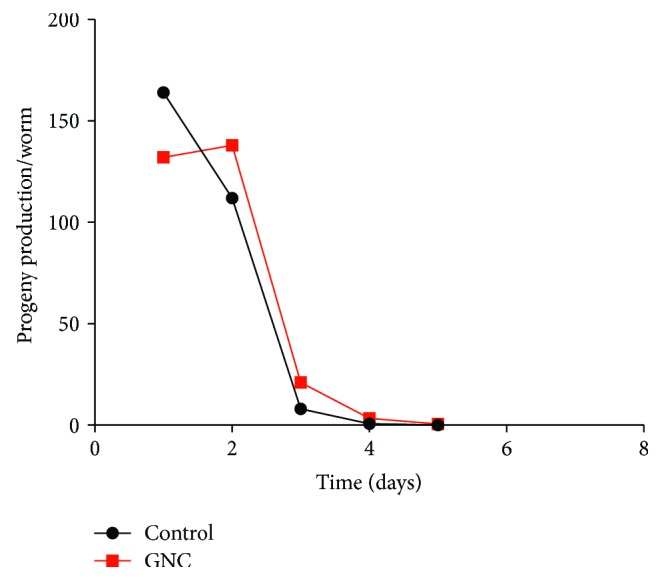
The effect of GNC on reproduction. Variation in the distribution of the reproductive capacity and total fecundity exhibited no significant difference between the GNC- (3.94 mg/mL) treated and untreated animals. A representative of three experiments with identical results is shown.

As reported, lifespan enhancement and stress resistance are often associated with a reduction in fecundity [16, 17]. In this study, we investigated the effect of GNC on *C. elegans* reproduction. We found that there was no significant difference in total progeny production between the GNC and control groups (Figure 3), indicating that GNC does not extend the lifespan of N2 worms by maintaining self-fertile reproduction at a slower rate.

### 3.4. Genome-Wide Transcriptional Profiling of GNC-Treated *C. elegans* N2

**Figure 4:**
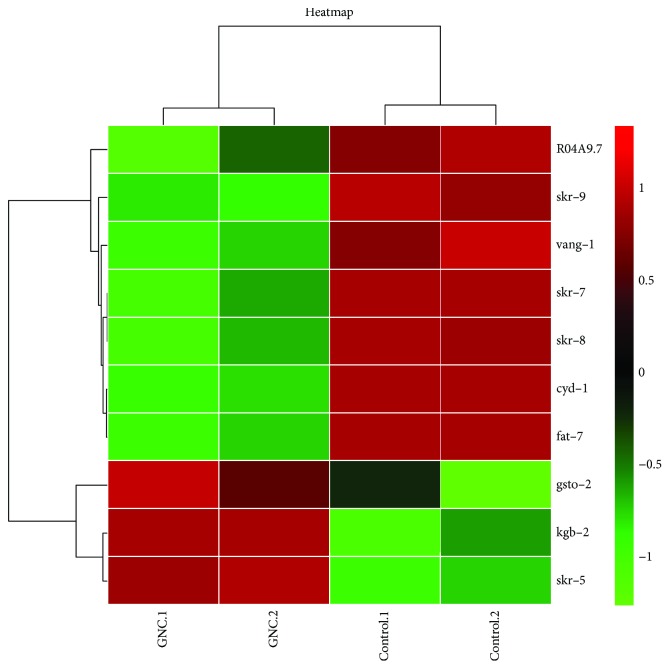
Hierarchical clustering of the 10 differentially expressed genes in 22-day-old worms treated with GNC (3.94 mg/mL) or controls. Each row represents a differentially expressed gene, while each column represents a sample. In the heatmap, green represents genes that are downregulated and red represents genes that are upregulated. Compared with the control group, the expressions of R04A9.7, skr-9, vang-1, skr-7, skr-8, cyd-1, and fat-7 genes were downregulated, while the expressions of gsto-2, kgb-2, and skr-5 genes were upregulated after GNC treatment (*p* < 0.05).

To identify a genetic contribution to GNC-mediated lifespan extension, we carried out RNA-seq analyses on the 22nd day to compare the transcriptional profiles of worms grown in the presence of GNC and controls. Differential gene expression in response to GNC treatment was assessed. We identified 10 different genes that may be associated with the prolongevity effect of GNC (Figure 4); compared with the control group, GNC downregulated the expressions of R04A9.7, skr-9, vang-1, skr-7, skr-8, cyd-1, and fat-7 genes, while it upregulated the expressions of gsto-2, kgb-2, and skr-5 genes (*p* < 0.05). The corresponding functional annotations of these genes are shown in Table 2 (GENE database was used for searching the functions of genes; https://www.ncbi.nlm.nih.gov/gene).

### 3.5. Validation of Differentially Expressed Genes Using qPCR

**Figure 5:**
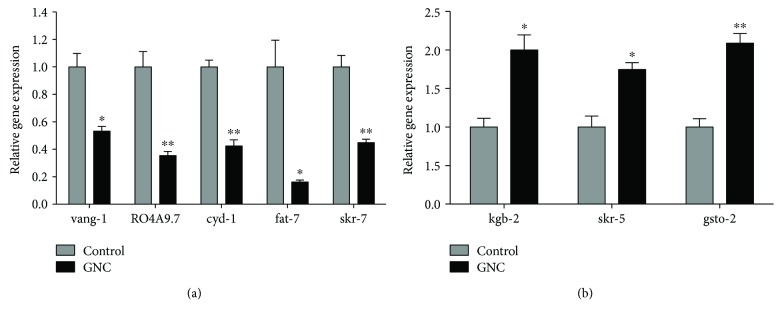
Validation of the differentially expressed genes screened by RNA-seq. (a) GNC (3.94 mg/mL) treatment downregulated the relative levels of the vang-1, R04A9.7, cyd-1, fat-7, and skr-7 transcripts. (b) GNC treatment upregulated the relative levels of the kgb-2, skr-5, and gsto-2 transcripts. The data are displayed as the mean ± SEM. ^∗^ *p* < 0.05; ^∗∗^ *p* < 0.01.

To validate the RNA-seq results, we performed qPCR analyses. The results showed that compared to control, the expressions of vang-1, R04A9.7, cyd-1, fat-7, and skr-7 genes were downregulated and the expressions of kgb-2, skr-5, gsto-2 genes were upregulated (*p* < 0.05). The eight genes each showed the same tendency as them observed in the RNA-seq experiments (Figure 5).

### 3.6. The Lifespan Extension Effect of GNC May Depend on the Insulin/IGF-1 Signalling Pathway

**Figure 6:**
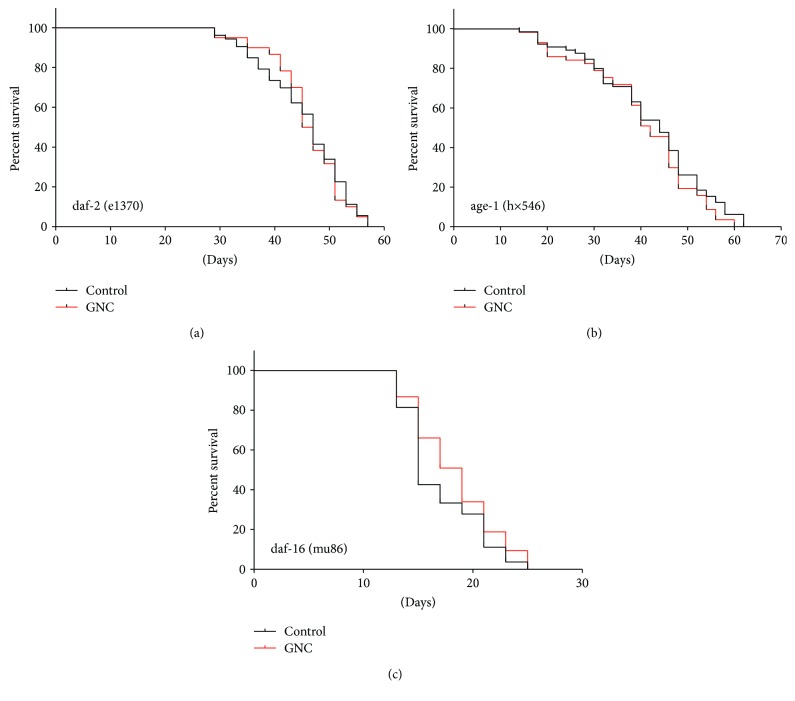
GNC extended the lifespan of *C. elegans* and may regulate the insulin/IGF-1 signalling pathway. Survival curves of the mutants (a) daf-2 (e1370), (b) age-1 (hx546), and (c) daf-16 (mu86) treated with control or GNC (3.94 mg/mL).

Among all the validated genes, one downregulated gene, VANG-1, was reported to modulate *C. elegans* lifespan via insulin/IGF-1-like signalling [18]. We further investigated whether GNC could interact with molecules in the insulin/IGF-1 signalling pathway to regulate lifespan. Our results showed that GNC failed to extend the longevity of the long-lived insulin-like receptor mutant daf-2 (e1370) (*p* > 0.05) (Figure 6(a)). In *C. elegans*, insulin-like ligands interact with DAF-2/insulin receptors to activate phosphoinositide 3-kinase age-1/PI3K and regulate the activity of multiple downstream targets, including the DAF-16/FOXO transcription factor [19]. Our results showed that GNC failed to increase the lifespan of age-1 (hx546) and daf-16 (mu86) mutant strains (*p* > 0.05) (Figures 6(b) and 6(c)), suggesting that the effect of GNC on lifespan extension was dependent of the age-1 and daf-16 genes.

### 3.7. The Lifespan Extension Effect of GNC Does Not Depend on Dietary Restriction or M-TOR and Germline Signalling Pathways

**Figure 7:**
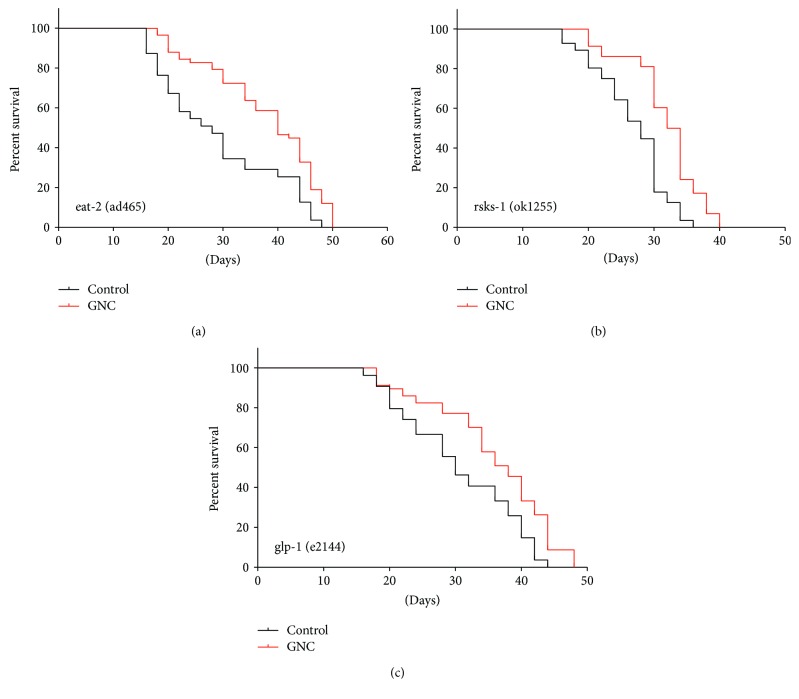
The effect of GNC on the longevity of characterized mutant strains. Notes: survival curves of the mutants (a) eat-2 (ad465), (b) rsks-1 (ok1255), and (c) glp-1 (e2144) treated with control or GNC (3.94 mg/mL).

In addition to the insulin/IGF-1 pathway, which regulates ageing, dietary restriction, the m-TOR pathway, and germline signalling pathway were extensively studied. In this study, we found that GNC could extend the lifespan of the mutants eat-2 (ad465), rsks-1 (ok1255), and glp-1 (e2144) (*p* < 0.05), suggesting that the longevity effect of GNC was independent of these pathways (Figures 7(a)–7(c)). Overall, we concluded that GNC extended the lifespan of *C. elegans* primarily via the insulin/IGF-1 signalling pathway.

## 4. Discussion

Ageing is a physiological process inherent to all living beings that results in a progressive decline in cellular protection and physiological functions. Ageing has deleterious effects on the health of human beings. Many modern diseases, such as cancer, diabetes, neuronal degeneration, and protein aggregation diseases, are ageing-related conditions [20]. Whether ageing may be prevented and/or postponed by certain approaches is a matter of utmost importance. TCM, along with other natural herbs, has received increased attention worldwide because of its long history regarding antiaging applications. For example, *Sargassum fusiforme* was shown to protect against oxidative stress during ageing [21]. More recently, Lutchman reported six plant extracts that slow yeast ageing more efficiently than any known chemical compound [22].

GNC, a classic TCM, has been found to improve learning and memory, delay skin ageing, and resist oxidative stress conditions [23, 24]. In our previous study, we reported that GNC significantly increased the *C. elegans* lifespan in a dose-dependent manner [7]. However, the mechanism through which GNC exerts this antiaging effect remains unclear. In this study, we used *C. elegans* as an in vivo model to investigate the antiaging effect of GNC. As shown in Figure 1(a), 3.94 mg/mL GNC significantly extended the lifespan of *C. elegans*, which is consistent with our previous research.

Ageing is a multifactorial process that is still poorly understood. Several theories have been proposed to explain the nature of ageing. One of the best known is the free radical theory of ageing by Harman, which identifies the free radicals produced by mitochondrial metabolism as the cause of cellular and DNA damage [25]. Consistent with this theory, increased reactive oxygen species (ROS) levels are frequently detected in aged tissues [26], suggesting that they are the major cause of ageing [27]. Oxidative stress is the disequilibrium between ROS and antioxidants [28]. Uncontrolled ROS production will eventually lead to accumulated damage of lipids, nucleic acids, proteins, and carbohydrates, causing cellular dysfunction and increasing susceptibility to harmful external agents [29]. In *C. elegans*, many studies have shown that enhancing resistance to oxidative stress could help extend lifespan [3, 21]. Therefore, we performed oxidative stress resistance assays. Our findings showed that the survival of wild-type worms under oxidative stress conditions induced by H_2_O_2_ or paraquat was significantly increased following GNC treatment; furthermore, GNC enhanced the heat resistance of *C. elegans*. From these results, we hypothesised that the lifespan extension effect may due to stress resistance mediated by GNC.

Next, in age-related phenotype experiments, we tested the chemotaxis behaviour and fecundity of *C. elegans*. Ageing is related to progressive declines in both motor function and muscle structure, which impair behavioural responses to sensory cues [15]. *C. elegans* has impressive chemotaxis behaviour [30]. In this study, the CI was used to evaluate the chemotaxis behaviours of the nematode *C. elegans*. As shown in Figure 2, chemotaxis behaviour was significantly improved after treatment with GNC (3.94 mg/mL), suggesting that GNC can improve health. In fecundity assays, GNC had no influence on total progeny production, suggesting that GNC did not extend the lifespan of *C. elegans* by reducing fecundity.

To analyse age-related changes in gene expression, we used an RNA-seq method. We set the 22nd day (corresponding to the old adult stage) as the time point to compare gene expression after GNC treatment. We identified 10 different genes that may be associated with the prolongevity effect of GNC (Table 2), and the qPCR results showed that eight genes each presented the same tendency as them observed in the RNA-seq experiments, with significant differences. Among these genes, VANG-1, the only *C. elegans* orthologue of the conserved planar cell polarity (PCP) component Strabismus/Van Gogh, has been reported to regulate lifespan via insulin/IGF-1-like signalling [18]. We wondered whether GNC extends lifespan through the insulin/IGF-1 signalling pathway, and thus, we conducted lifespan assays with a series of worm mutants.

Insulin/IGF-1 signalling is well known as the first pathway shown to regulate the lifespan of *C. elegans*. Furthermore, the insulin/IGF-1 signalling pathway plays an important role in stress resistance, metabolism, and reproduction [31]. Insulin-like ligands interact with DAF-2 [an orthologue of the mammalian insulin and insulin-like growth factor-1 (IGF-1) receptor] to activate AGE-1 [the *C. elegans* orthologue of phosphoinositide 3-kinase (PI3K)], thereby preventing daf-16 (FOXO) activation through Akt (AKT-1/2) stimulation [19]. In this study, mutant daf-2 (e1370), age-1 (hx546), and daf-16 (mu86) worms were tested. Our results showed that GNC failed to further extend the longevity of the mutant daf-2 (e1370), age-1 (hx546), and daf-16 (mu86) worms, suggesting that GNC extends the lifespan of *C. elegans* by regulating the insulin/IGF-1 signalling pathway. Many compounds, such as Withanolide A [32] and Chlorogenic Acid [4], exert the prolongevity properties in the same way.

Furthermore, we also wondered whether GNC could act on other signalling pathways. Dietary restriction (DR) prolongs the lifespan, which is conserved from yeast to mammals [33]. The eat-2 (ad465) II mutant is used as a DR model with pharyngeal pumping defects. In our study, GNC prolonged the lifespan of eat-2 (ad465) mutants, indicating that GNC might not act through a DR mechanism. mTOR signalling is a highly conserved pathway that regulates lifespan and cellular stress responses; rsks-1, which encodes the putative ribosomal protein S6 K, is required for protein synthesis [34]. We tested the survival time of mutant rsks-1 (ok1255) worms and found that GNC increased the lifespan of the rsks-1 mutants, which suggests that GNC did not act on the mTOR signalling pathway.

The germline of *C. elegans* also influences lifespan; glp-1, which encodes the receptor for a germline proliferation signal, regulates lifespan and oxidation in worms [35, 36]. We performed a lifespan assay using the mutant glp-1 (e2144) and found that GNC increased the lifespan, suggesting that the longevity effect of GNC was independent of the germline signalling pathway.

## 5. Conclusions

In summary, in this study, we found that GNC, consistent with its effect on age-related disease, increased the lifespan of *C. elegans* via the insulin/IGF-1 signalling pathway. Given the wide usage and the beneficial effect of GNC on human health, further tests in vertebrate models are merited.

## Figures and Tables

**Table 1:** Oligonucleotide primers used in qRT-PCR studies.

Gene	Forward primer	Reverse primer
act-4	GCCACCGCTGCCTCCTCATC	CCGGCAGACTCCATACCCAAGAAG
vang-1	GAAGGATTGGTGGATATGCTG	CAGTGTTCTGAACATGGGAG
R04A9.7	GGAGGTTACGGGAAGGTAT	ATGCCTCAAGTCTTTCTCAG
cyd-1	CATATTCAACTCGACATGCG	CGTGATGTTCTCCTGAACG
fat-7	CCATACGATACTTCTGTTTCCG	TGTAGTCTTGTGGGAATGTGT
skr-7	TCACTGTTCCAGAATGGGATAC	TGCATAGCTCATCAATCCAGG
kgb-2	CTGTTGACAATGAGTGCAAG	GAAGTATCCGGTGTGAAAGC
skr-5	CAAGAAAGTTATCGAATGGTGT	CATCCCATTCACCAATATCGTC
gsto-2	GATACAGCAGAGGAACTCTTG	GATATGGGAAGTCCAGAAAGC

**Table 2:** Summary of genes regulated by GNC (3.94 mg/mL) treatment on the 22nd day.

Gene ID	Gene symbol	Fold change	Description
180579	vang-1	0.45	Vang-like protein
3565956	R04A9.7	0.37	Hypothetical protein
174941	cyd-1	0.41	G1/S-specific cyclin-D
179100	fat-7	0.23	Delta(9)-fatty-acid desaturase fat-7
178840	skr-7	0.38	SKp1 related (ubiquitin ligase complex component)
178495	skr-8	0.33	SKp1 related (ubiquitin ligase complex component)
178494	skr-9	0.27	SKp1 related (ubiquitin ligase complex component)
191169	kgb-2	2.03	Mitogen-activated protein kinase
353420	gsto-2	2.14	Probable glutathione transferase omega-2
180148	skr-5	2.31	SKp1 related (ubiquitin ligase complex component)

## Supplementary Materials

Supplementary Materials Supplementary Table 1: composition and preparation of GNC. The exact proportion of each herb was shown.
