# Supplementary material for: Gengnianchun Extends the Lifespan of Caenorhabditis elegans via the Insulin/IGF-1 Signalling Pathway
Source: Oxid Med Cell Longev. 2018 Feb 18;2018:4740739. doi: 10.1155/2018/4740739 (PMC5835280; doi:10.1155/2018/4740739)
Supplement: Supplementary Materials — Supplementary Table 1: composition and preparation of GNC. The exact proportion of each herb was shown. [file 4740739.f1.docx]

**S. Table.1**: Composition and preparation of GNC

| TCM ID | Water extract(g) ()extracts extracts (g) |
| --- | --- |
| Radix Rehmanniae | 4.5 |
| Rhizoma Coptidis | 0.5 |
| Radix Paeoniae Alba | 1.2 |
| Rhizoma Anemarrhenae | 3.75 |
| Cistanche Salsa | 3.6 |
| Radix Morindae Officinallis | 3.6 |
| Poria | 0.9 |
| Epimedium Brevicornums | 0.6 |
| Cortex Phellodendri Amurensis | 0.75 |
| Fructus Lycii | 4.8 |
| Semen Cuscutae | 0.6 |
| Carapax et plastrum Testudinis | 0.75 |
